# Case Report: Extrapulmonary tuberculosis presenting as multiple caseous pericardial masses

**DOI:** 10.3389/fcvm.2025.1529400

**Published:** 2025-04-30

**Authors:** Juan Wang, Run Zhang, Zhengliang Li, Hui Fang, Wenzhong Zhang

**Affiliations:** Department of Cardiology, Affiliated Hospital of Qingdao University, Qingdao, Shandong, China

**Keywords:** extrapulmonary tuberculosis, pericardial multiple caseous masses, bloody pericardial effusion, pleural effusion, tuberculous pericarditis

## Abstract

The diagnosis of tuberculous pericarditis presenting as a hemorrhagic pericardial effusion is not difficult to make, but the presence of multiple pericardial masses in tuberculous pericarditis is uncommon. The article reports a 55-year-old Asian woman with a 10-day history of fever, chest tightness and shortness of breath. Laboratory investigations revealed an elevated C-reactive protein and erythrocyte sedimentation rate, and echocardiography showed a small amount of pericardial effusion associated with multiple pericardial caseous masses (up to approximately 2.4 cm × 6.9 cm) without pericardial constriction. Ten ml of bloody pericardial effusion was punctured and sent for pathology without malignant cells, and malignant mesothelioma was excluded in combination with PET-CT results. The diagnosis of extrapulmonary tuberculosis was finally confirmed by a positive Mantoux test and positive tuberculosis immunoreactivity, and the patient is now receiving standardized anti-tuberculosis treatment in a specialist hospital. Nowadays, the diagnosis of tuberculous pericarditis is not difficult, but the symptoms of a concomitant giant mass are rare, and its nature and treatment options (including drugs or surgery) are worth exploring.

## 1 Introduction

Tuberculosis (TB) is the leading cause of death from a single infectious disease. It is a global health problem that has been classified as a public health emergency for decades (1). Although the lung is the main site of primary infection with Mycobacterium tuberculosis, extrapulmonary tuberculosis is still involved in 20% of cases, with lymphadenitis being the most common site, followed by pleural tuberculosis. However, tuberculous pericarditis accounts for only 1%–4% of all cases (2, 3).

Tuberculous pericarditis usually implies reactivated disease and the site of primary infection may not be obvious. Pericardial caseous masses are very rare manifestations of tuberculosis. They may be confused with primary or metastatic pericardial tumors (4). This case illustrates the unusual presentation of extrapulmonary tuberculosis and was ultimately diagnosed as pericardial tuberculosis. The nature and treatment options of the pericardial mass were further explored.

## 2 Case presentation

A 55-year-old middle-aged woman with no history of chronic or infectious diseases presented to the respiratory medicine department with a 1-week history of intermittent fever and cough without runny nose, chest pain, chest tightness, breathlessness, blood in sputum, night sweats and weight loss. She had no known allergies and had never smoked or drunk alcohol.

On initial assessment, the patient was admitted with a regular respiratory rate, clear breath sounds in both lungs and no dry or wet rales. The heart rhythm was synchronous, the heart sound was slightly low and no obvious murmur was heard in any valve auscultation area. There was no lower limb oedema.

**Figure 1:**
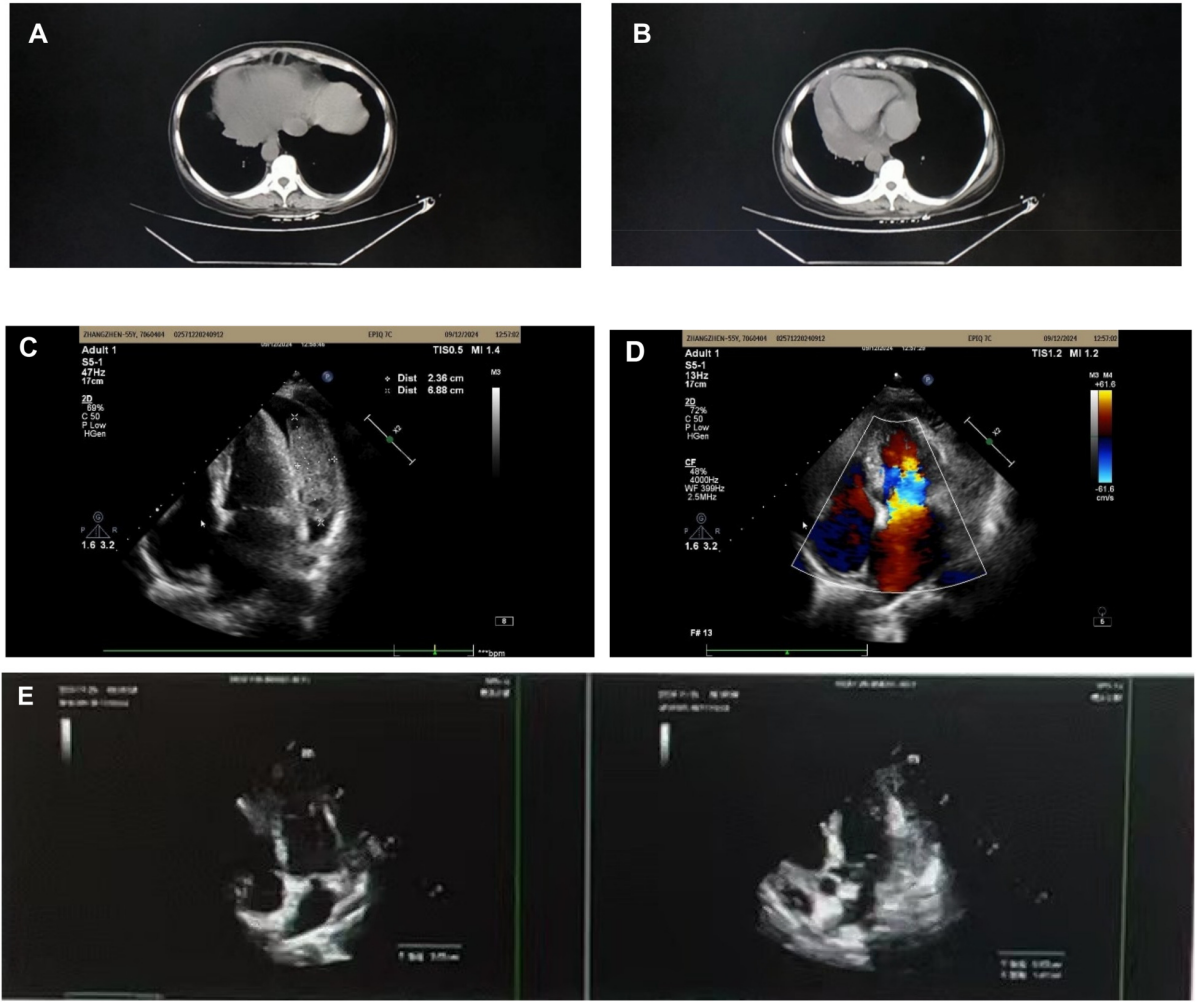
**(A)** Bilateral pleural effusion. **(B)** Pericardial effusion. **(C,D)** Pericardial effusion with multiple masses. **(E)** 6-month follow-up echocardiogram.

Complete blood count on admission showed no leukocytosis (6.38 × 103/μl), microcytic hypochromic anemia with hemoglobin of 9.6 g/dl, and c-reactive protein counts 243.5 mg/L. Other abnormal indicators include C-reactive protein 243.5 mg/L, procalcitonin 0.084 ng/ml, erythrocyte sedimentation rate 103 mm/60 min, and D-dimer 5,170 ng/ml. ANCA related indices, ENA enzyme profiling, COVID-19 test, A-flow and B-flow serologies were negative and tumor marker tests showed no obvious abnormality. Chest CT suggested a pericardial effusion associated with bilateral pleural effusions (Figures 1A,B), and further echocardiography was performed to identify a pericardial effusion with an irregular mass (Figures 1C,D). Levofloxacin and azithromycin were administered since the onset of fever with poor results. She was referred to cardiology for a definitive diagnosis. Considering neoplastic or tuberculous causes, PET-CT was performed to visualize multiple left pericardial masses with nodular soft tissue foci; bilateral pleural effusions. Malignant mesothelioma or infectious pericarditis could not be excluded.

The patient suddenly experienced chest tightness and difficulty breathing during hospitalization, accompanied by premature atrial contractions. Acute progression of pericardial effusion was suspected and symptomatic supportive care was given. Pericardiocentesis was performed the following day and 10 ml of non-coagulable fluid was removed; re-puncture was not performed due to the patient's pleural reaction and no malignant cells were found in the single puncture. Pleural irritation sign refers to an adverse reaction that occurs when the pleura is irritated. The three main features of pleural irritation sign are chest pain, chest tightness and dyspnoea. The patient complained of chest pain, chest tightness and dyspnoea, followed by a sudden onset of generalised convulsions. He was treated with epinephrine sedation, oxygen rehydration and other symptomatic supportive therapy. After puncture, the patient's symptoms improved and repeat echocardiography revealed multiple caseous masses in the pericardium (maximum size approximately 2.4 cm × 6.9 cm). A Mantoux test and a T-cell test for tuberculosis infection were performed to further clarify the diagnosis, and the results were positive, as shown in Table 1. The patient had a rapid onset of disease, combined with a large and multiple pericardial mass, and was considered to have rapidly progressive tuberculous pericarditis.

With the diagnosis of tuberculosis established, the patient was temporarily admitted to a specialist hospital for standardized treatment. Antituberculous therapy was started with rifampicin, isoniazid, pyrazinamide and ethambutol. In addition, adjuvant corticosteroid therapy was administered for one month (prednisone at an initial dose of 20 mg, then gradually tapered).

**Table 1:** T-cell test for tuberculosis infection from blood sample.

Project name	Result	Abnormality	Unit	Reference range
Culture/interferon (basic)	0.05		IU/ml	
Culture/interferon (stimulation)	0.42		IU/ml	
Culture/interferon (stimulation)	0.60		IU/ml	
Culture/interferon (positive)	2.54		IU/ml	
Interferon gamma release assay	0.37	↑	IU/ml	<0.35
Interferon gamma release assay	0.55	↑	IU/ml	<0.35
Positive control reaction	2.49		IU/ml	≥0.5
Tuberculosis immune response prompt	Positive	↑		

After treatment with hormones in combination with anti-tuberculosis drugs, the patient was examined with chest CT and cardiac ultrasound (Figure 1E), which showed that the pleural effusion had disappeared and several pericardial masses had been resorbed (approximate range 1.4*6.6) compared with the previous ones (approximate range 2.4*6.9).

## 3 Discussion

TB is a multi-system disease, and under-diagnosis of extrapulmonary TB is a challenge for TB control programs. Patients with EPTB do not always present with the typical symptoms of TB, such as chronic cough, sputum production, loss of appetite, weight loss, fever and night sweats. This makes it difficult to diagnose EPTB based on clinical symptoms alone (5). Tuberculosis may present as orbital tuberculosis with visual loss and headache (6), pancreatic tuberculosis with non-specific abdominal pain (7), or tuberculosis may invade the muscle tissue and cause primary purulent myositis (8). In our case, it was pericardial tuberculosis presenting as multiple pericardial caseous masses, bloody pericardial effusion and bilateral pleural effusions.

Pericardial tuberculosis is a rare oligobacillary manifestation of extrapulmonary tuberculosis. Because of its oligobacillary nature, it is difficult to diagnose by laboratory tests alone and requires invasive procedures such as pericardiocentesis (9). However, for pericardial TB, the highest detection rates appear to come from pericardial biopsy (10%–64%) and TB PCR of pericardial tissue (80%), which is limited by its invasive nature, while culture of TB from pericardial fluid has a detection rate of (53%–75%) (10). Unstimulated interferon γ (IRISA-TB) is more sensitive than the Mantoux test for the diagnosis of TB pericarditis in TB-poor settings (11). Our case was also definitively diagnosed by the interferon γ-related test.

Otherwise, pericardial masses due to tuberculosis are extremely rare. Clinically, pericardial masses are usually attributed to malignant tumors, and metastatic pericardial involvement is more common than primary tumors and is usually associated with a poor prognosis (12). To date, the pathophysiology of tuberculous pericardial masses is not clear; it has been suggested that they are due to aggregation of red blood cells and proteinaceous material. It has been suggested that these masses may be associated with amyloidosis (13). Echocardiography can detect pericardial caseous masses to some extent, but the nature of these is not clear. In recent years, cardiac magnetic resonance has been shown to have significant advantages over echocardiography in the detection, characterization and assessment of cardiovascular abnormalities (14), and may become the “mainstay” of diagnosis of pericardial TB. New diagnostic modalities are also emerging and there has been a case of tuberculous pericarditis diagnosed by transgastric EUS fine needle aspiration in a foreign country (15).

In cases of rapidly progressive TB pericarditis such as the one described above, studies have shown that a high proportion of patients treated with medication and pericardiocentesis alone will progress to constrictive pericarditis, which can seriously affect the patient's prognosis. Early surgical intervention (pericardiocentesis or pericardiotomy) can significantly reduce the likelihood of rapidly progressive TB pericarditis progressing to constrictive pericarditis, thus improving the overall prognosis of patients, the quality of their survival and reducing the burden on society (16).

We will analyse the uniqueness of the case in terms of imaging manifestations of the pericardial caseous mass, diagnosis of exclusion (diagnostic imaging and therapeutic diagnosis) and short-term hormonal shock therapy for acute progressive pericarditis.

### 3.1 Imaging of pericardial caseous mass

Pericardial tuberculosis usually presents with pericardial effusion, pericardial thickening or constrictive pericarditis as the main imaging manifestations, but the imaging characteristics of this patient are peculiar, showing multiple inhomogeneous masses suggesting caseous necrosis. This presentation is rare in tuberculous pericarditis and there is imaging overlap with some malignancies (e.g., pericardial mesothelioma or metastatic pericardial tumours).

### 3.2 Rule-out diagnosis

#### 3.2.1 Imaging diagnosis

To exclude malignancy, PET-CT was performed without significant hypermetabolic foci, and no malignant cells were detected in the pericardial effusion; to exclude other bacterial, fungal, or viral infectious diseases, relevant pathogenicity tests were performed without positive end results; and to exclude immune-mediated diseases, autoimmune index tests were performed without abnormalities.

#### 3.2.2 Therapeutic diagnosis

When histological evidence could not be obtained, patients were rationally treated with antituberculosis therapy, and the efficacy was evaluated by imaging follow-up. This also suggests that anti-tuberculosis drug therapy may still be effective in certain patients who are not suitable for surgery.

### 3.3 Short-term hormonal shock therapy for acute progressive pericarditis

In some cases of tuberculous pericarditis, there is a risk of rapid exacerbation of pericardial inflammation or even pericardial tamponade due to an overreaction of the immune system. In this case, the patient received hormonal shock therapy at the time of exacerbation, mainly to reduce the acute inflammatory response and prevent further deterioration of the pericarditis. However, hormones must be used with caution, especially in the context of tuberculosis infection, as they can suppress the immune system and interfere with the efficacy of anti-tuberculosis therapy.

This case highlights a case of tuberculosis occurring in an atypical site and presenting with atypical symptoms, and provides a differential diagnosis to consider for similar symptoms of unknown cause, such as pericardial caseous masses, hemorrhagic pericardial effusions and bilateral pleural effusions.

How clinicians can suspect and confirm tuberculous pericarditis in atypical cases? Suspicion and confirmation of tuberculous pericarditis in atypical cases requires a combination of history, clinical presentation, laboratory tests and imaging studies. Risk factors include a history of previous TB exposure, immunocompromise and epidemiological background, while patient symptoms such as low-grade fever, chest tightness and shortness of breath, and slowly progressive pericardial effusion should also raise red flags. Pericardiocentesis is an important early diagnostic tool, analysis of the effusion can suggest a high-protein exudate, elevated adenosine deaminase (ADA) can support a diagnosis of TB, and pathogenetic testing (PCR or culture) can help confirm the diagnosis. Imaging is crucial in the differential diagnosis; echocardiography may suggest an effusion, CT/MRI may show pericardial thickening or calcification, and PET-CT may be used to rule out malignancy. In this case, PET-CT showed no hypermetabolic lesions, which, combined with the absence of detectable tumour cells on examination of the pericardial effusion, made tuberculous pericarditis a highly suspect diagnosis. As the patient did not undergo a pericardial biopsy, the diagnosis was ultimately supported by imaging changes following antituberculosis treatment. This case demonstrates that in the absence of histological evidence, a combination of imaging, pericardial fluid analysis and response to treatment can provide strong diagnostic support for tuberculous pericarditis and highlights the value of PET-CT in differentiating tuberculosis from malignant lesions.

## 4 Conclusion

The case of an Asian woman with multiple caseous pericardial masses due to tuberculosis is presented. In patients presenting with these symptoms and an unknown diagnosis, the possibility of tuberculosis involvement should be considered to ensure a clear diagnosis and timely initiation of systemic anti-tuberculosis therapy; delays may lead to the development of chronic constrictive pericarditis or drug resistance. The nature of the mass and treatment options are also discussed.

## Data Availability

The original contributions presented in the study are included in the article/supplementary material, further inquiries can be directed to the corresponding author.
